# Transmission pathways and spillover of an erythrocytic bacterial pathogen from domestic cats to wild felids

**DOI:** 10.1002/ece3.4451

**Published:** 2018-09-11

**Authors:** Annie Kellner, Scott Carver, Valeria Scorza, Clifton D. McKee, Michael Lappin, Kevin R. Crooks, Sue VandeWoude, Michael F. Antolin

**Affiliations:** ^1^ Graduate Degree Program in Ecology Colorado State University Fort Collins Colorado; ^2^ Department of Biology Colorado State University Fort Collins Colorado; ^3^ Department of Fish, Wildlife and Conservation Biology Colorado State University Fort Collins Colorado; ^4^ School of Biological Sciences University of Tasmania Hobart Tasmania Australia; ^5^ Department of Clinical Sciences Colorado State University Fort Collins Colorado; ^6^ Department of Microbiology, Immunology, and Pathology Colorado State University Fort Collins Colorado

**Keywords:** ancestral state reconstruction, *Ca. Mycoplasma haemominutum*, cross‐species transmission, disease ecology, host‐shift

## Abstract

Many pathogens infect multiple hosts, and spillover from domestic to wild species poses a significant risk of spread of diseases that threaten wildlife and humans. Documentation of cross‐species transmission, and unraveling the mechanisms that drive it, remains a challenge. Focusing on co‐occurring domestic and wild felids, we evaluate possible transmission mechanisms and evidence of spillover of *“Candidatus Mycoplasma haemominutum*” (*CMhm*), an erythrocytic bacterial parasite of cats. We examine transmission and possibility of spillover by analyzing *CMhm* prevalence, modeling possible transmission pathways, deducing genotypes of *CMhm* pathogens infecting felid hosts based on sequences of the bacterial 16S rRNA gene, and conducting phylogenetic analyses with ancestral state reconstruction to identify likely cross‐species transmission events. Model selection analyses suggest both indirect (i.e., spread via vectors) and direct (i.e., via interspecific predation) pathways may play a role in *CMhm* transmission. Phylogenetic analyses indicate that transmission of *CMhm* appears to predominate within host species, with occasional spillover, at unknown frequency, between species. These analyses are consistent with transmission by predation of smaller cats by larger species, with subsequent within‐species persistence after spillover. Our results implicate domestic cats as a source of global dispersal and spillover to wild felids via predation. We contribute to the emerging documentation of predation as a common means of pathogen spillover from domestic to wild cats, including pathogens of global conservation significance. These findings suggest risks for top predators as bioaccumulators of pathogens from subordinate species.

## 1 INTRODUCTION

It is widely acknowledged that the majority of known pathogens infect multiple host species; for example, 90% of pathogens infecting domestic dogs and cats infect other mammalian hosts (Cleaveland, Laurenson, & Taylor, 2001). Yet documentation of spillover events, and an understanding of their underlying factors, remains a major challenge. For spillover to occur, multiple barriers must be overcome, including a suite of intra‐ and interspecific, environmental, and within‐host processes (Plowright et al., 2017). Pathogen spillover may include dead‐end events, where the pathogen fails to persist in the new host species, or persistence with onward transmission and establishment in the new host species (a “host‐shift”; Lloyd‐Smith et al., 2009; Longdon, Brockhurst, Russell, Welch, & Jiggins, 2014; Vicente‐Santos et al., 2017). Important predictors of pathogen spillover include phylogenetic relatedness, geographic overlap, and interspecific interactions among host species (Olival et al., 2017). Anthropogenic landscapes, with their complex wildlife–livestock–human interfaces, can create novel opportunities for pathogens to cross species (Hassell, Begon, Ward, & Fèvre, 2017).

The genetic, physiological, and physical proximity of domestic and wild felids facilitates their shared susceptibility to many of the same infectious agents (Bevins et al., 2012; O'Brien & Yuhki, 1999). These qualities, in addition to the globally ubiquitous presence of domestic cats in urban settings, render felids an excellent system for the study of pathogen spillover at the wildland–urban interface. Haemotropic mycoplasmas (“haemoplasmas”)—a group of bacterial, erythrocytic parasites widely distributed in felids (Tasker, 2010; Willi et al., 2005)–are likely to spill over between species. The haemoplasmas are dominated by three species: the most common being “*Candidatus Mycoplasma haemominutum*” (*CMhm*), followed by *Mycoplasma haemofelis* and “*Candidatus Mycoplasma turicensis*.” The relative prevalence of the latter two varies among species, and the prevalence of all are likely underestimated (Barker & Tasker, 2013; Willi, Filoni, et al., 2007b). Disease from haemoplasma infection ranges from subclinical to severe chronic and acute anemia, depending on the mycoplasmal species and the underlying health of the host (Luria et al., 2004; Reynolds & Lappin, 2007; Willi et al., 2005). Compelling genetic evidence suggests domestic cats may be the source of the global distribution of multiple strains of haemoplasmas in wild felids, and high prevalence among some wild felids (Willi, Filoni, et al., 2007b) may also suggest persistent onward transmission following spillover.

Haemoplasma transmission (intra‐ or interspecific) is poorly understood. Initially, transmission was presumed to be vector‐borne because of phenotypic similarity to vector‐borne rickettsia (Neimark, Johansson, Rikihisa, & Tully, 2001) and because of co‐occurrence of mycoplasmas in tick and flea vectors and in blood of their domestic hosts (Barrs et al., 2010; Fyumagwa et al., 2008; Shaw, Kenny, Tasker, & Birtles, 2004; Woods, Brewer, Hawley, Wisnewski, & Lappin, 2005). Laboratory infection experiments, however, have been inconclusive, and vector‐borne transmission results in only transient infections of *M. haemofelis* (Woods, Wisnewski, & Lappin, 2006). *CMhm* DNA has been isolated from the saliva of domestic cats and vampire bats, implicating social grooming and aggression as possible mechanisms of direct transmission (Dean, Helps, Jones, & Tasker, 2008; Volokhov et al., 2017). Museux et al. (2009) simulated social transmission and transmission by aggression by infecting domestic cats with “*Ca. M. turicensis*” subcutaneously, orally, and oronasally. Of these, only subcutaneous inoculations with infected blood resulted in successful transmission. Thus, the mechanisms of transmission for this closely related group of pathogens, and their relative importance in determining prevalence, remain unresolved.

Understanding pathogen spillover and mechanisms of transmission has conservation implications for wild felids. Other high‐profile (nonhaemoplasma) spillover events from domestic to wild cats have caused major population declines among wild felids, including an outbreak of feline panleukopenia virus in bobcats in 1988 (Wassmer, Guenther, & Layne, 1988) and outbreaks of feline leukemia (FeLV) in Iberian lynxes in 2006 (Meli et al., 2009) and Florida Panthers in 2008 (Brown et al., 2008). Additionally, nonfatal cross‐species infections of feline immunodeficiency virus have occurred between bobcats and pumas (Franklin et al., 2007; Lee et al., 2014, 2016), and feline gammaherpesvirus from bobcats has been identified as the predominant subtype in pumas (Troyer et al., 2014). It is possible that predator‐driven pathogen exposure is the common mechanism of cross‐species transmission underlying these events (Lee et al., 2016). Interspecific killing and predation among felids are well‐documented, with larger cats typically killing smaller ones (Cashman, Peirce, & Krausman, 1992; Koehler & Hornocker, 1991; Palomares & Caro, 1999). Recent studies confirm that pumas periodically prey on domestic cats, particularly in areas with high housing densities (Smith, Wang, & Wilmers, 2016; Kevin Blecha, pers.comm., 3/31/16). The spillover among domestic and nondomestic felids has likely been more frequent than previously reported, and spillover also may be common among other taxa, such as canids. By studying specific parasites such as *CMhm* a more general understanding of shared forms of transmission among different pathogens/parasites may emerge.

This study evaluates spillover of *CMhm* in domestic and wild felids, including assessment of transmission mechanisms and evidence of onward transmission. We analyze field surveys of sympatric domestic cats, bobcats, and pumas from multiple sites in the United States in conjunction with global genetic data on *CMhm* in felids. The analyses connect field surveys, modeling of possible transmission pathways, sequencing of the bacterial 16S rRNA gene found in host blood, and Bayesian phylogenetic analyses with ancestral state reconstruction. We hypothesize that *CMhm* is primarily host‐specific and transmitted through direct contact, with occasional cross‐species transmission. We predict patterns of cross‐species transmission of *CMhm* will support predation as a mechanism for pathogen spillover from domestic to wild cats and demonstrate onward transmission in wild felid species following spillover.

## 2 METHODS

### 2.1 North American study populations

**Figure 1:**
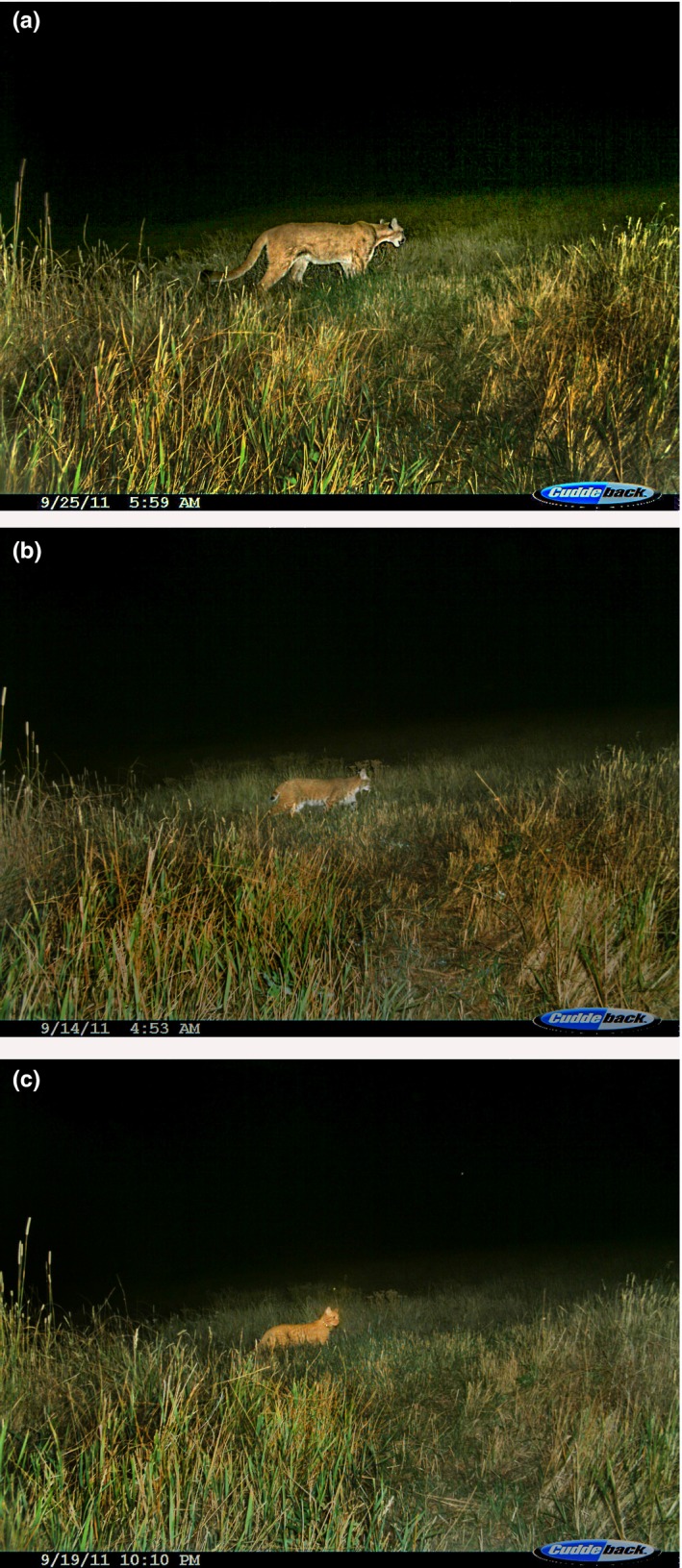
Camera trap footage of spatiotemporal overlap of a puma (a), bobcat (b), and domestic cat (c). Photographs taken at the same trapping location in September 2011, on the wildlife–urban interface adjacent to Boulder, CO, during an affiliated camera trap study

**Figure 2:**
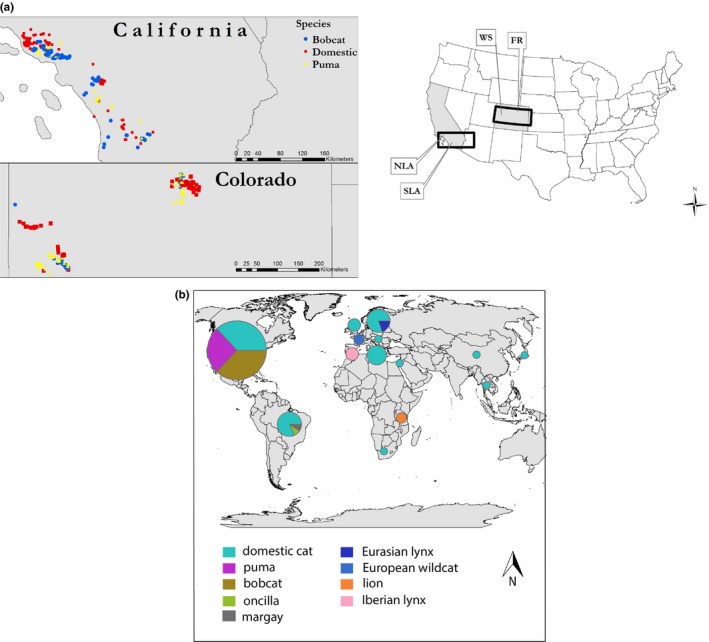
(a) North American capture locations of pumas, bobcats, and domestic cats. California locations include Ventura and Los Angeles counties north of the City of Los Angeles (NLA), and Orange, San Diego, and Riverside counties south of the City of Los Angeles (SLA). Colorado capture locations include the Uncompahgre Plateau on the Western Slope (WS) of the Rocky Mountains near the city of Montrose and the Front Range (FR) east of the Rocky Mountains near the city of Boulder. (b) Geographic origins of all host species from which CMhm isolates were used for phylogenetic analyses (Supporting Information Appendix S3). Samples include novel isolates from North American felids (*n* = 60) as well as previously described sequences from GenBank (*n* = 49). The size of the pie chart is scaled to the number of samples included from each region (largest = 65; smallest = 1)

We sampled sympatric pumas (*Puma concolor*), bobcats (*Lynx rufus*), and domestic cats (*Felis catus*) from four sites in California and Colorado (Figures 1 and 2a). California sites comprised two regions, north (NLA) and south (SLA) of the city of Los Angeles. Colorado sites included rural areas on the Western Slope (WS) of the Rocky Mountains near the cities of Montrose and Grand Junction, and the Front Range (FR) of the Rocky Mountains, immediately adjacent to Boulder, CO. Capture locations ranged from urbanized landscapes to natural areas. Samples were collected through multiple studies between the years 2001 and 2012. For more details on study locations and sampling methods, see Carver et al. (2016).

Estimates of species‐ and site‐specific densities (animals per km^2^) were obtained from published reports (Table 1). Of the three felid species studied, densities of the domestic cat were least well‐known. Where they were not known for the specific study site, general estimates of urban edge and rural densities were compiled from published studies across the United States (Table 1).

**Table 1:** Occurrence of *CMhm* infection (*I*) among feral domestic cat, bobcat, and puma samples (*N*) from southern California and Colorado as determined by PCR detection of 16S rRNA sequences. Also shown are proportion of males (*m*, estimated from sample collection) and species densities (*d*, per km^2^, estimated from literature). *N*,* I*,* m* and *d* were used in transmission pathway models (see Table 2)

Site	Species	*N*	*I* (%)	*m*	*d*
North Los Angeles	Domestic	74	22 (29.7)	0.6	35.2^a^
California	Bobcat	179	85 (47.5)	0.5	0.21^b^
	Puma	32	20 (62.5)	0.7	0.008^c^
South Los Angeles	Domestic	56	8 (14.3)	0.5	35.2^a^
California	Bobcat	20	13 (65.0)	0.8	0.23^b^
	Puma	10	5 (50.0)	0.7	0.011^c^
Western Slope Colorado	Domestic	59	9 (15.3)	0.5	9.1^d^^,^ e ^,^ f
	Bobcat	25	8 (32.0)	0.6	0.194^g^
	Puma	46	21 (45.7)	0.4	0.022^g^
Front Range	Domestic	56	3 (5.4)	0.5	35.2^a^
Colorado	Bobcat	15	7 (46.7)	0.7	0.192^g^
	Puma	59	38 (64.4)	0.4	0.032^g^

a Dabritz, Atwill, Gardner, Miller, and Conrad (2006).

b Riley, Boydston, Crooks, and Lyren (2010).

c Beier, Riley, and Sauvajot (2010).

d Warner (1985).

e Coleman and Temple (1993).

f Hubbs (1950).

g Lewis et al. (2015).

### 2.2 *CMhm* infection and prevalence

Infection with *CMhm* was initially assessed from DNA extracted from red blood cells and characterized through conventional PCR assays targeting the 16S rRNA gene, following the protocol established by Jensen, Lappin, Kamkar, and Reagan (2001). This protocol is sensitive and specific to mycoplasma species and has been used extensively in studies of feline haemoplasmas (e.g., Bergmann et al., 2017; Lobetti & Lappin, 2012; Luria et al., 2004). In total, we tested 631 samples (147 pumas, 239 bobcats, 245 domestic cats), of which 239 tested PCR‐positive for *CMhm* (Table 1). To test for effects of host species, sex, and site on the probability of CMhm infection, we used logistic regression. We summarize the samples screened for *CMhm* infection and the major population characteristics used for modeling of transmission mechanisms in Table 1.

### 2.3 Modeling of transmission pathways

To evaluate factors relating to intra‐ and interspecific *CMhm* transmission among felids, we developed algebraic expressions describing possible transmission pathways, categorized by host species and incorporating species‐ and site‐specific parameters derived from the literature and/or determined by the authors (Tables 1 and 2). Intraspecific transmission through social contact (1a, Table 2) assumes transmission by social interactions (e.g., grooming) and low‐level aggressive encounters that may result from social interactions. Social contact hypotheses may include transmission by host‐specific arthropod vectors, but evidence suggests this is unlikely for all three felid species (Currier, 1983). “Sex effects” (1b, Table 2) include interactions influenced by the proportion of males in the host population, such as male‐dominated territorial or aggressive encounters (e.g., Drewe 2010). For interspecific transmission, we assumed intraguild predation routes could only occur from smaller to larger species, that is, predation of domestic cat by bobcat or puma and predation of bobcat by puma only (2a–d, Table 2). Vector‐borne transmission was assumed to take place via generalist vectors that could include ticks, fleas, or mosquitoes (3a, Table 2). We also assumed the possibility of some directionality in vector‐borne transmission, that is, bobcats and puma having host‐specific or shared vectors and acquiring infected host‐specific vectors from domestic cats, domestic cats acquiring host‐specific vectors from bobcats and pumas, and domestic cats acquiring shared vectors from bobcats and pumas (3b–e, Table 2). For environmental transmission (3f, Table 2), we assumed species‐specific acquisition of environmental fomites from items such as substrate, carcasses, prey, and scat (for scent/territory marking).

**Table 2:** Algebraic expressions representing possible pathways of CMhm transmission

Transmission pathways	Transmission to			
	(1) Domestic cat	(2) Bobcat	(3) Puma	(4) Puma (domestic cat predation only)
1. Intraspecific pathways				
1a. Social contact	*θ*_sc*D*_ *d*_*D*_	*θ*_sc*B*_ *d*_*B*_	*θ*_sc*P*_ *d*_*P*_	
1b. Sex effect	*θ*_sx*D*_ *m*_*D*_ *d*_*D*_	*θ*_sx*B*_ *m*_*B*_ *d*_*B*_	*θ*_sx*P*_ *m*_*P*_ *d*_*P*_	
2. Interspecific pathways: predation				
2a. P predate D				*θ*_pr*PD*_ (*I*_*D*_/*N*_*D*_) *d*_*D*_
2b. B and P predate D		*θ*_pr*BD*_ (*I*_*D*_/*N*_*D*_) *d*_*D*_		*θ*_pr*PD*_ (*I*_*D*_/*N*_*D*_) *d*_*D*_
2c. P predate B and D			*θ*_pr*PB*_ (*I*_*B*_/*N*_*B*_) *d*_*B*_	*θ*_pr*PD*_ (*I*_*D*_/*N*_*D*_) *d*_*D*_
2d. B and P predate D and P predate B		*θ*_pr*BD*_ (*I*_*D*_/*N*_*D*_) *d*_*D*_	*θ*_pr*PB*_ (*I*_*B*_/*N*_*B*_) *d*_*B*_	*θ*_pr*PD*_ (*I*_*D*_/*N*_*D*_) *d*_*D*_
3. Intra‐ and Interspecific pathways: vector and environmental				
3a. Generalist vector	*θ*_v_ *d*_*D*_	*θ*_v_ *d*_*B*_	*θ*_v_ *d*_*P*_	
3b. B and P acquire D vectors	*θ*_v*D*_ *d*_*D*_	(*θ*_v*D*_+*θ*_v*B*_)*d*_*B*_	(*θ*_v*D*_+*θ*_v*P*_)*d*_*P*_	
3c. B and P share vectors and acquire D vectors	*θ*_v*D*_ *d*_*D*_	(*θ*_v*D*_+*θ*_*BP*_)*d*_*B*_	(*θ*_v*D*_+*θ*_v*BP*_)*d*_*P*_	
3d. D acquire B and P vectors	(*θ*_v*D*_+*θ*_v*B*_+*θ*_v*P*_)*d*_*D*_	*θ*_v*B*_ *d*_*B*_	*θ*_v*P*_ *d*_*P*_	
3e. D acquire shared B and P vectors	(*θ*_v*D*_+*θ*_v*BP*_)*d*_*D*_	*θ*_v*BP*_ *d*_*B*_	*θ*_v*BP*_ *d*_*P*_	
3f. Environmental	*θ*_e*D*_ (*d*_*D*_+*d*_*B*_+*d*_*P*_)	*θ*_e*B*_ (*d*_*D*_+*d*_*B*_+*d*_*P*_)	*θ*_e*P*_ (*d*_*D*_+*d*_*B*_+*d*_*P*_)	

Notes

Combinations of these expressions form a priori hypotheses that were fit to observed prevalence data and ranked using AICc (see Tables 1 and 3, and Appendix S1). Estimated parameters represented by *θ*; each unique subscript of *θ* represents an independently estimated parameter.

*D*: domestic cat; *B*: bobcat; *P*: puma; *N*: number of samples tested (Table 1); *I*: number of infected samples (Table 1); *d*: site‐specific density of species per km^2^ (Table 1); *m*: proportion of samples that are male (Table 1); *θ*_sc_: transmission from social contact; *θ*_sx_: sex effect; *θ*_pr_: transmission from predation; *θ*_v_: vector‐borne transmission; *θ*_e_: environmental transmission.

Because multiple transmission pathways may determine observed *CMhm* prevalence among species and sites, we combined these algebraic expressions by adding or subtracting as appropriate to form a priori models (Tables 2 and 3, Supporting Information Appendix S1). Models were ranked according to their fit to observed prevalence (I/N, Tables 1 and 2) among species and sites in California and Colorado (Table 1). Our models are simplifications of possible transmission pathways, given the limited information available on haemoplasma ecology and contact networks among felids. Seasonal‐ and age‐specific differences in infection were not modeled because sample sizes were too small to evaluate this confidently for some species at some sites. We did not use susceptible‐infected‐recovered (SIR) or other forms of ordinary differential equation models for these same reasons.

**Table 3:** Model selection of best fit (∆AICc < 5) a priori hypotheses for intra‐ and interspecific *CMhm* transmission (*I*/*N*)

A priori hypothesized transmission model	Model number	K	−2LOG(L)	AICc	ΔAICc	Weight (%)	Cumulative weight (%)
Generalist vector (3a)	12	1	87.998	90.398	0	34.9	34.895
Sex effect (1b), generalist vector (3a)	17	4	78.859	92.573	2.176	11.8	46.651
Sex effect (1b), B and P share vectors and acquire D vectors (3c)	19	4	78.859	92.573	2.176	11.8	58.408
D acquire shared B and P vectors (3e)	16	2	87.529	92.862	2.465	10.2	68.583
P predate D (2a), generalist vector (3a)	22	2	87.995	93.328	2.931	8.1	76.644
B and P predate D (2b), generalist vector (3a)	23	3	86.11	95.11	4.72	3.3	79.944

Note

See Table 2 for algebraic expressions (denoted in parentheses) and parameter definitions. Model number refers to a priori hypotheses listed in Supporting Information Appendix S1.

Fit of a priori models to species‐ and site‐specific prevalence data and estimation of unknown parameters (represented by the generic symbol *θ*, with subscripts denoting single and multiple parameter estimates) were calculated using maximum likelihood, based on binomial error distributions. Thetas (*θ*) encompass cumulative information for the felid populations, such as contact rates, transmission probabilities, seasonality of dynamics, environmental determinants of dynamics, and vector abundance and species composition (for *θ*_v_). These scaling variables are necessary to define and parameterize these models/hypotheses, but their values are not immediately biologically meaningful in and of themselves. Each unique subscript of theta represents an independently estimated parameter.

For each species‐specific component, we applied an inverse logit transformation to constrain prevalence estimates between zero and one. Fixed parameters include animal density (*d*) and proportion of males (*m*). Prevalence (*I*/*N*) of prey species is also used as a fixed parameter within interspecific transmission models; specifically, the fixed prevalence of the prey species at a given site is a variable influencing the estimation of prevalence in the predator species at the same site. Best‐fitting models were then identified using model selection based on Akaike's information criterion corrected for small sample size (AICc). All analyses were conducted in R (R Core Team 2017) using the stats and stats4 packages.

### 2.4 Genotyping by sequencing of *CMhm* isolates

Of the 239 *CMhm*‐positive samples, we sequenced the 16S rRNA gene in a subset of 82 individuals for our phylogenetic analyses. We excluded animals co‐infected with multiple *Mycoplasma* species (e.g., infected with both *CMhm* and *M. haemofelis*; Supporting Information Appendix S2) and preferentially selected samples from which DNA had been extracted for prior studies. We aimed for a roughly equal distribution of pumas, bobcats, and domestic cats from each North American study area. We ultimately excluded samples that failed to amplify, resulted in unreadable sequences, or were co‐infected with >2 unique *CMhm* genotypes. Our final data set of *CMhm* sequences included 73 novel *CMhm* sequences from North American felids (19 domestic cats, 24 bobcats, and 17 pumas; Supporting Information Appendix S3). Thirteen North American individuals were co‐infected, with two 16S sequences detected in the same samples. In addition, we incorporated 49 previously described sequences from GenBank for domestic cats (*n* = 38) and wild felid species (*n* = 12) (Figure 2b; Supporting Information Appendix S3).

Our final alignment of directly sequenced samples consisted of high‐quality, unambiguous reads of ≥2× coverage, sequenced in both directions. We trimmed all sequences to an equal length of 1,238 nucleotides, including insertion–deletion mutations (indels). Our final alignment (excluding the outgroup) included 159 variable positions of which 103 were informative (i.e., the same nucleotides shared by two or more haplotypes). To generate phylogenetic trees and determine evolutionary relationships among *CMhm* genotypes, we used the BEAUTi graphical user interface for program BEAST version 1.8.4 to input parameters for Bayesian Markov chain Monte Carlo (MCMC) analyses (Drummond & Rambaut, 2007; Drummond, Suchard, Xie, & Rambaut, 2012), making use of the BEAGLE library to improve computational performance (Suchard & Rambaut, 2009). We assumed a strict molecular clock, and checked this assumption by running trees with unconstrained branch lengths, including neighbor‐joining, unweighted pair group method with arithmetic mean (UPGMA), and maximum‐likelihood trees to confirm the repeatability of our topology. Further details on PCR amplification, sequencing, detection of co‐infections, and phylogenetic analyses can be found in Supporting Information (Appendices S4 and S5).

### 2.5 Ancestral state reconstruction

We further examined our best‐fitting phylogenetic trees (a) to test whether including the felid host or geographic origin of a *CMhm* sample better explained the evolutionary relationship between *CMhm* isolates, (b) to estimate median pairwise transition rates and associated support between all host‐to‐host and location‐to‐location combinations, and (c) to identify the most likely ancestral host at each branching point along our tree. Following protocols for ancestral state reconstruction (Faria, Suchard, Rambaut, Streicker, & Lemey, 2013; Hayman, McDonald, & Kosoy, 2013; Lemey, Rambaut, Drummond, & Suchard, 2009; McKee, Hayman, Kosoy, & Webb, 2016; Streicker et al., 2010), we assigned discrete traits to each *CMhm* genotype based on host species (“host”) and geographic origin (“location”). For all ancestral state analyses, we established a prior of a diffuse gamma distribution with shape and scale parameters set to one (see Supporting Information Appendix S6 for a complete list of priors). We ran marginal likelihood analyses on symmetric and asymmetric diffusion models, evaluating model performance by marginal likelihood estimation (MLE) based on path and stepping‐stone sampling (Baele et al., 2012). We also quantified phylogeny–trait correlations using two statistics described by Parker, Rambaut, and Pybus (2008) (association index and parsimony score) to ascertain whether models involving host species or geographic origin better fit relationships between *CMhm* genotypes. For geographic origin, we tested both country (e.g., United States) and region (e.g., North America). We used the software package BaTS for all calculations (Parker et al., 2008) and ran 1,000 simulations for each of 1,000 trees.

Host‐to‐host transition rates and counts were estimated by comparing 10,000 possible phylogenetic trees (Table 5, Figure 6). We used an asymmetrical model for reconstructing host‐to‐host transition events because of our interest in assessing the variation in these rates. Transition rates were measured in transitions per substitution per site, meaning the rate is measured continuously in molecular time, and are reported as medians of the posterior distributions over all sampled trees. As an additional means of measuring host‐to‐host transitions, we used a robust counting procedure to estimate the number of Markov jumps (i.e., discrete host transitions) across all branches of all resulting trees (Faria et al., 2013; O'Brien, Minin, & Suchard, 2009). We assessed the weight of the evidence for these measurements using a Bayesian stochastic search variable selection (BSSVS) procedure (Faria et al., 2013; Lemey et al., 2009). We applied a calculation from Lemey et al. (2009) in which the Bayes factor (BF) for a given transition rate *k* equals the posterior odds that *k* is nonzero divided by its prior odds,

$${\text{BF}}_{k}=\frac{p_{k}/\left(1-p_{k}\right)}{q_{k}/\left(1-q_{k}\right)},$$

where *p*_*k*_ is the posterior probability that rate *k* is nonzero, and *q*_*k*_ is the prior probability. Because we employed an asymmetrical model of diffusion, the Poisson prior on the rates connecting *K* host species has a mean of *K*−1 and a zero offset, with twice as many rates as a symmetrical model. Thus, *q*_*k*_ is calculated as follows (Lemey 2016, personal communication):

$$q_{k}=\frac{K-1}{K(K-2)}.$$

We considered BF > 3 as supportive of an estimated transition rate, BF > 10 as strongly supportive, BF > 30 as very strongly supportive, and BF > 100 as decisive (Faria et al., 2013).

The ancestral host assigned to each node is represented as a state probability derived from the posterior distribution. When both the posterior probability and the state probability of a node were high (>0.90), we concluded there was strong support for the hypothesis that a given branching event occurred and that the host depicted at the node was the true ancestral host at that particular node in the tree.

## 3 RESULTS

### 3.1 Prevalence

**Figure 3:**
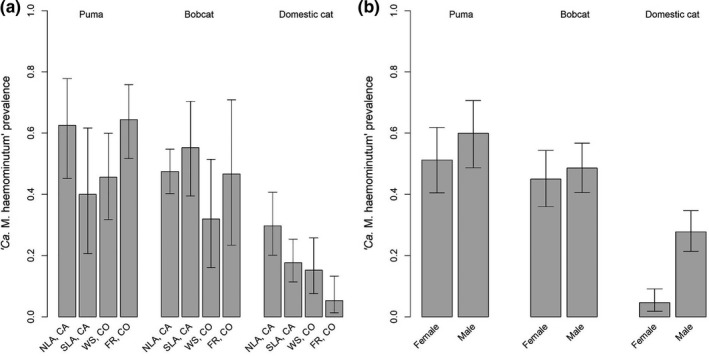
Prevalence (±95% CIs) of *CMhm* among puma, bobcat, and domestic cat, delineated by (a) site and (b) sex

Infection prevalence was highest in pumas (range: 0.46–0.63) and bobcats (range: 0.32–0.65) and generally lower in domestic cats (range: 0.06–0.30) across our study sites in Colorado and California (Table 1; Figure 3). Wild felids (pumas and bobcats) and males (all species pooled) were more likely to test positive for *CMhm* than domestic or female cats. We found no differences in prevalence among study sites (Table 4).

**Table 4:** Effect of host species, sex, and location on *CMhm* infection, from logistic regression analysis

	Coef.	*SE*	*Z*	*p*
Bobcat	1.282	0.214	6.002	<0.001
Puma	1.910	0.238	8.024	<0.001
Male	0.618	0.173	3.583	<0.001
NLA	0.341	0.263	1.298	0.194
SLA	0.047	0.283	0.167	0.867
WS	−0.274	0.287	0.954	0.340

Note

Coefficients relative to domestic cat, females, and front range, CO (FR).

### 3.2 Modeling of transmission pathways

We modeled combinations of intra‐ and interspecific pathways of *CMhm* transmission and assessed the likelihood of these models relative to observed prevalence across felids and study sites (Supporting Information Appendix S1). We observed model uncertainty, with both vector and direct‐transmission pathways appearing in the top models (Table 3). Generalist vector (3a, Table 2) was the top model, with 34.9% of the model weight. Sex effect (1b, Table 2), predation (2a, 2b, Table 2), and shared vectors (3c, 3e, Table 2) also appeared in the best fit models.

**Figure 4:**
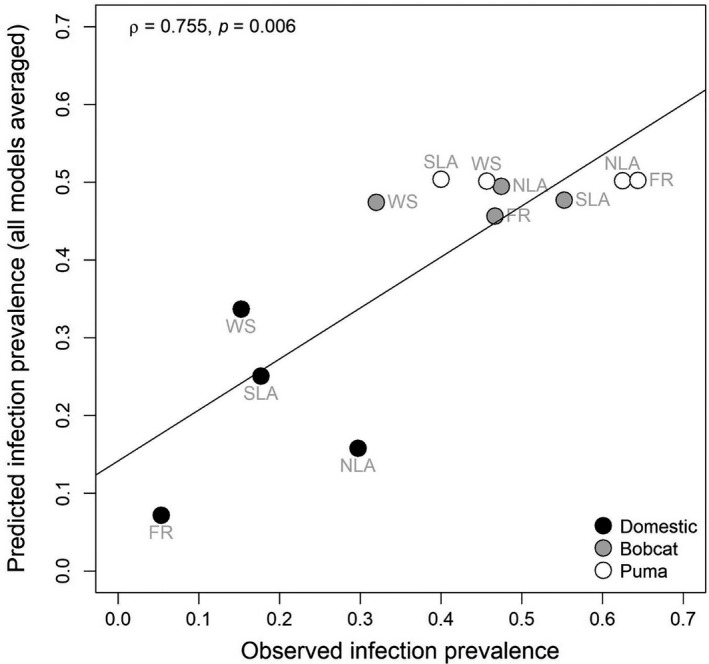
Relationship between observed *CMhm* prevalence of infection (*I*/*N*) to model‐averaged prevalence of infection. Spearman correlation *ρ* = 0.755; *p* = 0.006

We evaluated the fit of our models by correlating observed site‐ and species‐specific infection prevalence with the model‐averaged estimated site‐ and species‐specific infection prevalence across the entire model set. We found a moderately strong correlation between predicted and observed infection prevalence (Spearman correlation = 0.755; *p* = 0.006; Figure 4).

### 3.3 Phylogenetics and cross‐species transmission

**Figure 5:**
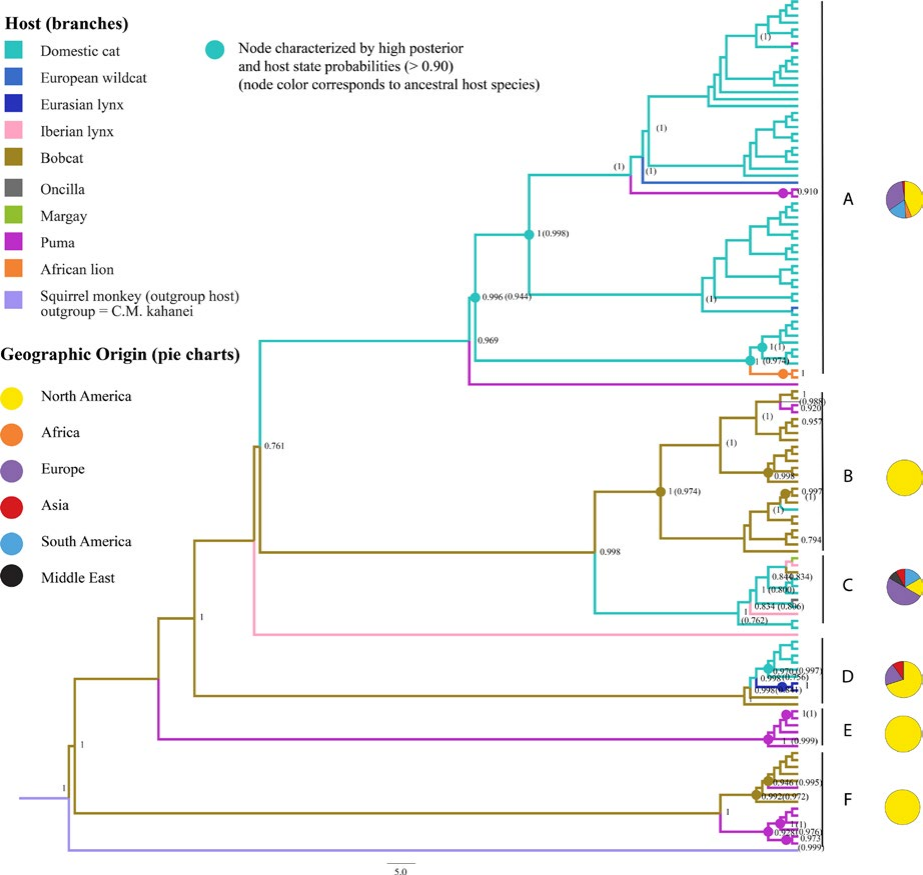
Bayesian phylogeny and ancestral host state reconstruction of *CMhm* genotypes. Node labels without parentheses indicate posterior probabilities (PP). Node labels within parentheses indicate posterior host state probabilities (SP). PP values and SP values >0.75 were indicated. To improve readability, we did not denote SP for nodes depicting very recent branching events, though all carried a SP ≥0.9, with the exception of the uppermost branching event in clade C (SP = 0.441). Circles indicate nodes in which both PP and SP probabilities >0.90, indicating strong support for both the branching event and the estimated ancestral host species. Circle coloration corresponds to host species. Clades are assigned letters A–F for reference. Pie charts correspond to the geographic origin of isolates analyzed in this study

Analysis of *CMhm* Bayesian tree topology revealed clustering patterns consistent with higher levels of intraspecific than interspecific transmission (Figure 5). Neighbor‐joining, UPGMA, and maximum‐likelihood trees confirmed this topology (Supporting Information Appendix S7). With a few exceptions, *CMhm* lineages largely cluster together within host species for pumas, bobcats, and domestic cats. For all other felids represented by sequences from GenBank, sample size was insufficient to verify whether *CMhm* genotypes were species‐specific (Figure 5). However, isolates drawn from pumas, bobcats, and domestic cats were also grouped within clusters of genotypes that originated from one of the other hosts, suggesting that both cross‐species transmission and host switching has occurred in the past (see Discussion).

Isolates from California and Colorado did not group together phylogenetically for either wild or domestic cat samples, visually affirming our empirical analysis of phylogeny–trait correlation. Calculations of association index (AI) and parsimony score (PS) using asymmetrical diffusion models and regional geographic origins (e.g., North America) revealed that the felid host (“host”) from which *CMhm* samples originated better explained our tree topology than their geographic origin (“origin”; AI host = 1.06; AI origin = 1.43; PS host = 20.68; PS origin = 23.73). Using symmetrical diffusion models and countries of origin yielded equivalent results (AI host = 1.03; AI region = 1.62; PS host = 20.73; PS origin = 23.96). The support for asymmetrical versus symmetrical diffusion models was equivocal for both host (path sampling BF = 1.69, stepping‐stone BF = 1.96) and location (path sampling BF = 0.64, stepping‐stone BF = 0.51) analyses.

Statistical support for particular branch points in the tree was mixed, with both recent splits and older clades supported. Despite a preponderance of host specificity in our trees, congruency between *CMhm* genotypes and the felid family tree was low in all clades but one (clade E, Figure 5). Isolates derived from larger, predator species nested within clades consisting primarily of their smaller prey, a pattern consistent with infrequent cross‐species transmission from prey to predators. An exception is seen in Clade B, where a domestic cat sequence assorts into a bobcat clade. The only divergent, species‐specific clade includes only puma hosts (clade E, Figure 5), consistent with the assumption that transmission would primarily be unidirectional from more subordinate hosts to dominant species.

### 3.4 Phylogenetic reconstruction of global cross‐species transmission events

**Figure 6:**
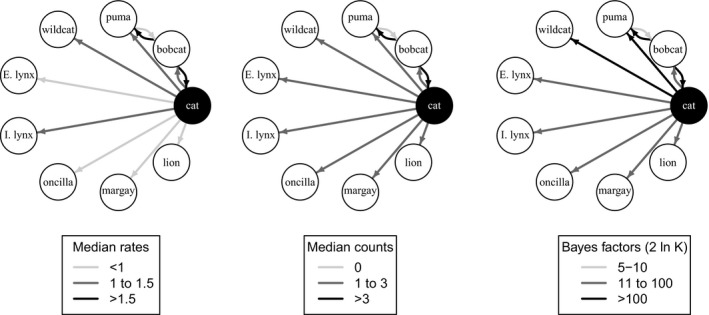
Median transition rates and counts between felid species with Bayes factor >3. “Rates” refer to the state‐to‐state transition rate per substitution per site over all trees, and “counts” refer to the number of Markov jumps (discrete host state changes) over all trees. Arrows indicate directionality of transmission

Of the 12 well‐supported host‐to‐host transitions (BF > 3), eight were from domestic to wild cats. Two originated in bobcats and spread to pumas and domestic cats, and one originated in pumas and transitioned to bobcats. One of the transitions reflects the initial branching of the outgroup into the *CMhm* tree (Table 5). Transitions from puma to bobcat, and from the outgroup to bobcats, were never observed and had relatively low BF support. This likely reflects that an ancestral host at these nodes was an unsampled host species. All domestic‐to‐wild transitions were highly to decisively supported (BF range: 29.08–1,043.96), as were bobcat‐to‐puma and bobcat‐to‐domestic cat transitions (BF 781 and 445, respectively; Table 5, Figure 6).

**Table 5:** Median transition rates and counts associated with host‐to‐host changes over all trees. Rates, counts, and 95% highest probability density (HPD) derived from the posterior distribution

Transition	Median rate	Rate 95% HPD interval	Median count	Count 95% HPD interval	Bayes factor
Cat → wildcat	1.08	[8.1E‐2, 2.79]	2	[2, 3]	1,043.96
Cat → Eurasian lynx	0.67	[2.6E‐3, 2.37]	1	[0, 2]	14.39
Cat → Iberian lynx	1.33	[1.1E‐2, 3.24]	3	[0, 4]	43.47
Cat → bobcat	1.05	[3.7E‐4, 3.19]	1	[0, 6]	17.87
Cat → oncilla	0.69	[5.5E‐3, 2.29]	1	[0, 2]	29.08
Cat → margay	0.70	[3.5E‐4, 2.29]	1	[0, 2]	17.95
Cat → puma	1.39	[8.0E‐2, 3.37]	3	[1, 5]	709.28
Cat → lion	0.67	[1.0E‐3, 2.23]	1	[0, 2]	30.37
Bobcat → puma	1.63	[2.3E‐1, 3.82]	4	[1, 6]	781.00
Bobcat → cat	1.80	[2.1E‐1, 4.14]	5	[1, 8]	445.50
Puma → bobcat	0.82	[1.7E‐4, 2.82]	0	[0, 4]	7.09
Outgroup → bobcat	0.64	[4.5E‐5, 2.65]	0	[0, 1]	4.09
All transitions	25.24	[13.56, 39.18]	27	[22, 33]	–

Phylogenetic analyses provide strong posterior support (>0.90) across many branch points in the tree in which the domestic cat was determined to be the original host of *CMhm* (clades A and C, Figure 5). Notably, these clades include *CMhm* isolates from disparate hosts and geographic locations (e.g., lions in Africa and margays in Brazil), indicating dispersal and diversification of *CMhm* on a global scale (Figure 5). In contrast, *CMhm* clades originating in puma or bobcat hosts show minimal geographic spread, but do show evidence of cross‐species transmission (e.g., Figure 5: clades B, E, and F include genotypes found only in North America; B and F show cross‐species transmission). We also observe what appears to have been a host switch and divergence of *CMhm* from bobcats to pumas (clade F, Figure 5).

## 4 DISCUSSION

In this study, we gained insight into cross‐species pathogen transmission, and potential for spillover and cross‐species host‐shifts, by combining models of alternative transmission pathways of *CMhm* and models of host origins via phylogenetic analyses based upon bacterial 16S rRNA gene sequences. In sum, we conclude that intraspecific transmission of *CMhm* may occur through either direct transmission or vectors, that cross‐species transmission and host‐shifts likely occur via predation of smaller cats by larger cats, and that domestic cats may be spreading haemoplasmas to their rarer, wild relatives on a global scale.

### 4.1 Prevalence

Prevalence of *CMhm* varied widely among populations, species, and geographic areas, in this study and others (Barker & Tasker, 2013; Willi, Filoni, et al., 2007b). Prevalence of *CMhm* in bobcats and pumas in our study sites was higher than in domestic cats and fell within the range of *CMhm* prevalence reported in a previous study of other wild cats not including North American felids (0.10–0.96, Willi, Filoni, et al., 2007b). Willi, Filoni, et al. (2007b) further differentiated between free‐ranging and captive wild individuals, noting that the prevalence of *CMhm* in free‐ranging animals (0.54) was much higher than in zoo‐born (0.05) or wild‐caught (0.16) captive animals. Our puma and bobcat samples were from free‐ranging cats and are comparable to the overall prevalence reported in free‐ranging wild cats (Willi, Filoni, et al., 2007b). *CMhm* prevalence for domestic cats in our study was similar to those found in previous studies (0.08–0.47, see Barker & Tasker, 2013), with the exception of the Western Slope group, where prevalence was slightly lower (0.06). Our study also suggests that male cats are more likely than female cats to test positive for *CMhm*; this effect of sex is likely strongest in domestic cats.

Our 0.035 co‐infection rate of *CMhm* with other haemoplasma species (i.e., *M. haemofelis* or “*Ca. M. turicensis*”) was also similar to previous studies (range: 0.001–0.13, mean 0.028; see Barker & Tasker, 2013). However, we also found a high rate of co‐infection of multiple *CMhm* genotypes, with >0.40 (34/82) of all samples co‐infected. This estimate is likely low, as we suspect many of the samples with poorly resolved sequence data included multiple genotypes of *CMhm* representing multistrain co‐infections.

### 4.2 Modeling of transmission pathways

Transmission dynamics of emerging and newly recognized pathogens are often unclear, particularly when wildlife hosts are concerned. This uncertainty confounds modeling efforts and impedes effective management (Tompkins, Dunn, Smith, & Telfer, 2011; Woodroffe et al., 2016). The natural route of haemoplasma transmission has not yet been identified, as previous studies concluded that either vector‐borne or direct transmission is the likely pathway (e.g., Shaw et al., 2004; Woods et al., 2006). The results of our model selection reinforce this uncertainty, as both vector‐borne and direct pathways appear in our top models, suggesting that multiple transmission pathways may exist concurrently. Our phylogenetic analysis supports the inclusion of direct pathways in our top model set by showing predation by dominant predators to be a driver of cross‐species transmission (see below). On the other hand, our phylogenetic analysis cannot provide inference on intraspecific transmission routes. Thus, it is possible that predation and vectors differentially link individuals within and among species.

### 4.3 Phylogenetics and reconstruction of cross‐species transmissions

Our phylogenetic analyses imply that predation by dominant predators is an important mechanism of cross‐species transmission. First, several genotypes sampled from wild felids share high sequence identity and clustered with isolates derived from their prey. Second, our ancestral state analyses show strong support for the prey species as the ancestral host for these clusters. Finally, genotypes from pumas comprise a divergent and species‐specific clade, which may reflect that pumas are the apex predator among the sampled North American felids, and rarely transmit their haemoplasmas to the smaller cats they prey upon. These results support the hypothesis by Willi, Filoni, et al., 2007b that domestic and wild cats may share haemoplasmas; we verify this exchange through our phylogenetic analysis and suggest predation as a contributing mechanism. We suspect our inability to reject symmetrical models (i.e., that isolates are exchanged bidirectionally with equal frequency between predator and prey species) likely stems from an absence of samples supporting certain directions of transmission (e.g., unlikely directions such as puma‐to‐domestic cat). While it is possible that predator and prey species exchange isolates via blood‐feeding arthropod vectors, the results from our phylogeny and ancestral state reconstruction show this is unlikely given the evidence supporting unidirectional transmission. Another reason for our inability to reject these models may simply be a weaker signal with respect to cross‐species transmission, as cross‐species transmission is rarer, making it difficult to accurately estimate its rate. In the instance where a domestic cat isolate falls into a bobcat clade (i.e., bobcat‐to‐domestic cat transmission), we cannot differentiate between vector‐borne exchange and direct transmission, as bobcats and domestic cats may interact aggressively without resultant mortality.

The emergent phylogenetic pattern of *CMhm* genotypes in pumas and bobcats is similar to that of feline immunodeficiency virus (FIV; Franklin et al., 2007; Lee et al., 2016) and gammaherpesvirus (GHV; Troyer et al., 2014), viral infections of North American felids transmitted through blood‐to‐blood contact. In recent phylogenetic analyses of these pathogens, several genotypes of each virus exist, with one specific to pumas and another shared between pumas and bobcats (Lee et al., 2016; Troyer et al., 2014). The similarities between phylogenetic patterns of FIV, GHV, and *CMhm* provide further support both for host switching by feline pathogens via predation events and for blood‐to‐blood contact as a transmission mechanism. Furthermore, we see strong directional support for bobcat‐to‐puma transmission in two instances based on our analysis of ancestral states and the high rates and counts of bobcat‐to‐puma host‐state transitions. Pumas are known to prey on bobcats, so this pattern is consistent with direct transmission of *CMhm* and viruses via predation (Cashman et al., 1992; Koehler & Hornocker, 1991). The high transition rate may reflect a long period of co‐existence and interaction between bobcats and pumas. Our methodology identifies bobcats as the ancestral host of all *CMhm* genotypes, but this result has low support. Wild felids outside North America are not sufficiently represented in this analysis to unambiguously identify the ancestral host of all *CMhm*. It is possible the true ancestral host of *CMhm* is an extinct felid species. Similarly, we used a single, highly conserved gene (16S rRNA) for phylogenetic analyses. The addition of sequence data from other loci and extensive sampling of wild cats outside North America would better define species‐specific clades, identify incidences of homologous recombination (particularly in co‐infections), and provide greater posterior support for nodes across the tree. Such efforts would elucidate the evolutionary origins of the feline haemoplasmas and provide further resolution of transmission pathways.

### 4.4 Conclusion

Wild felids experience multiple stressors, including habitat degradation (Schipper et al., 2008; Wilcove, Rothstein, Dubow, Phillips, & Losos, 1998), range restriction and fragmentation (Crooks, 2002; Crooks, Burdett, Theobald, Rondinini, & Boitani, 2011; Gaona, Ferreras, & Delibes, 1998), poaching (Kenney, Smith, Starfield, & McDougal, 1995), persecution (Inskip & Zimmermann, 2009), prey depletion (Karanth & Stith, 1999), climate change (Parmesan & Yohe, 2003), and restricted population size that may result in inbreeding depression (O'Brien & Yuhki, 1999; Roelke et al., 1993). Predator‐driven pathogen exposure, as documented in this study, may represent an underappreciated ecological driver of disease (Lee et al., 2016). Our results strongly support the domestic cat as the recent ancestral host of the most geographically and taxonomically diverse clades of *CMhm*. This result suggests that as domestic cats accompany humans across the globe, they concomitantly may lead to spillover of haemoplasmas into their wild counterparts. Even though some *CMhm* infections carry no obvious signs of disease, evidence suggests *CMhm* may in fact be a primary pathogen in felids (i.e., the causative agent of anemia; Reynolds & Lappin, 2007). Indeed, other haemoplasmas are of concern to wild populations. *Mycoplasma haemofelis*, for example, can cause severe hemolytic anemia in domestic cats, particularly when paired with comorbid retroviral infection (Luria et al., 2004); like *CMhm* and *M. haemofelis*, isolates found in domestic cats are closely related to those found in wild cats. Also concerning is the possibility that *CMhm* might be illuminating a pathway through which more virulent pathogens are transmitted. Under already compromised circumstances, the introduction of infectious disease into small populations of wild felids may become more common and more harmful.

## CONFLICT OF INTEREST

The authors declare no competing interests.

## AUTHOR CONTRIBUTIONS

Conceived and designed study: AK, SC, KRC, SV, and MFA. Procured samples: SC, KRC, SV, and ML. Conducted laboratory work: AK and VS. Contributed laboratory materials/space/support: VS and ML. Analyzed data: AK, SC, CDM, and MFA. Wrote paper: AK and SC. Critically revised paper: AK, SC, KRC, and MFA. Approved final version: all authors.

## DATA ACCESSIBILITY AND ARCHIVING

All model code, input files, genetic sequences, and supporting information have been archived in Dryad (http://datadryad.org). DOI: https://doi.org/10.5061/dryad.8rb517m
