# Supplementary material for: Transmission pathways and spillover of an erythrocytic bacterial pathogen from domestic cats to wild felids
Source: Ecol Evol. 2018 Sep 11;8(19):9779–92. doi: 10.1002/ece3.4451 (PMC6202716; doi:10.1002/ece3.4451)
Supplement: Supplementary file 1 [file ECE3-8-9779-s001.docx]

**SUPPLEMENTAL INFORMATION**

**Appendix S1.** *A priori* models of intra- and interspecific *CMhm* transmission pathways fit to observed infection prevalence (*I*/*N*), where the number of infected individuals (*I*) is represented by the following species-specific combination of expressions (*I_D_=*domestic; *I_B_=*bobcat; *I_P_*= puma). See Table 2 for expressions and parameter definitions. Model fit based on Akaike Information Criteria corrected for small sample size (AICc).

| Transmission Model # | | *I_D_* | *I_B_* | *I_P_* | K | -2LOG(*L*) | AICc | ∆AICc | Weight (%) |
| --- | --- | --- | --- | --- | --- | --- | --- | --- | --- |
| *Social contact* | |  |  |  |  |  |  |  |  |
| 1 | | 1a_1_ | 1a_2_ | 1a_3_ | 3 | 86.91 | 95.91 | 5.51 | 2.2 |
| *Sex effect* | | |  |  |  |  |  |  |  |
| 2 |  | 1b_1_ | 1b_2_ | 1b_3_ | 3 | 93.77 | 102.77 | 12.37 | 0.1 |
| *Social contact + Sex effect* | | |  |  |  |  |  |  |  |
| 3 | | 1a_1_+1b_1_ | 1a_2_+1b_2_ | 1a_3_+1b_3_ | 6 | 77.55 | 106.35 | 15.95 | 0.0 |
| *Social contact + Predation* | | |  |  |  |  |  |  |  |
| 4 | | 1a_1_-2a_4_ | 1a_2_ | 1a_3_+2a_4_ | 4 | 88.71 | 102.43 | 12.03 | 0.1 |
| 5 | | 1a_1_-2b_4_-2b_2_ | 1a_2_+2b_2_ | 1a_3_+2b_4_ | 5 | 86.47 | 106.47 | 16.08 | 0.0 |
| 6 | | 1a_1_-2c_4_ | 1a_2_-2c_2_ | 1a_3_+2c_4_+2c_2_ | 5 | 84.86 | 104.86 | 14.466 | 0.0 |
| 7 | | 1a_1_-2d_4_-2d_2_ | 1a_2_+2d_2_-2d_3_ | 1a_3_+2d_4_+2d_3_ | 6 | 81.92 | 110.72 | 20.33 | 0.0 |
| *Social contact + Sex effect + Predation* | | | |  |  |  |  |  |  |
| 8 | | 1a_1_+1b_1_-2a_4_ | 1a_2_+1b_2_ | 1a_3_+1b_3_+2a_4_ | 7 | 77.72 | 119.72 | 29.32 | 0.0 |
| 9 | | 1a_1_+1b_1_-2b_4_-2b_2_ | 1a_2_+1b_2_+2b_2_ | 1a_3_+1b_3_+2b_4_ | 8 | 74.89 | 138.89 | 48.50 | 0.0 |
| 10 | | 1a_1_+1b_1_-2c_4_ | 1a_2_+1b_2_-2c_2_ | 1a_3_+1b_3_+2c_4_+2c_2_ | 8 | 73.92 | 137.92 | 29.32 | 0.0 |
| 11 | | 1a_1_+1b_1_-2d_4_-2d_2_ | 1a_2_+1b_2_+2d_2_-2d_3_ | 1a_3_+1b_3_+2d_4_+2d_3_ | 8 | 75.24 | 139.24 | 48.84 | 0.0 |
| *Vector-borne* | |  |  |  |  |  |  |  |  |
| 12 | | 3a_1_ | 3a_2_ | 3a_3_ | 1 | 88.00 | 90.40 | 0.00 | 34.9 |
| 13 | | 3b_1_ | 3b_2_ | 3b_3_ | 3 | 86.96 | 95.96 | 5.56 | 2.2 |
| 14 | | 3c_1_ | 3c_2_ | 3c_3_ | 2 | 91.64 | 96.98 | 6.58 | 13.0 |
| 15 | | 3d_1_ | 3d_2_ | 3d_3_ | 3 | 86.55 | 95.55 | 5.15 | 2.7 |
| 16 | | 3e_1_ | 3e_2_ | 3e_3_ | 2 | 87.53 | 92.86 | 2.46 | 10.2 |
| *Vector-borne + Sex effect* | | |  |  |  |  |  |  |  |
| 17 | | 3a_1_+1b_1_ | 3a_2_+1b_2_ | 3a_3_+1b_3_ | 4 | 78.86 | 92.57 | 2.18 | 11.8 |
| 18 | | 3b_1_+1b_1_ | 3b_2_+1b_2_ | 3b_3_+1b_3_ | 6 | 74.90 | 103.70 | 13.31 | 0.0 |
| 19 | | 3c_1_+1b_1_ | 3c_2_+1b_2_ | 3c_3_+1b_3_ | 4 | 78.86 | 92.57 | 2.18 | 11.8 |
| 20 | | 3d_1_+1b_1_ | 3d_2_+1b_2_ | 3d_3_+1b_3_ | 6 | 74.80 | 103.60 | 13.20 | 0.0 |
| 21 | | 3e_1_+1b_1_ | 3e_2_+1b_2_ | 3e_3_+1b_3_ | 5 | 78.63 | 98.63 | 8.23 | 0.6 |
| *Vector-borne + Predation* | | |  |  |  |  |  |  |  |
| 22 | | 3a_1_-2a_4_ | 3a_2_ | 3a_3_+2a_4_ | 2 | 87.99 | 93.33 | 2.93 | 8.1 |
| 23 | | 3a_1_-2b_4_-2b_2_ | 3a_2_+2b_2_ | 3a_3_+2b_4_ | 3 | 86.11 | 95.11 | 4.72 | 3.3 |
| 24 | | 3a_1_-2c_4_ | 3a_2_-2c_2_ | 3a_3_+2c_4_+2c_2_ | 3 | 87.22 | 96.22 | 5.82 | 1.9 |
| 25 | | 3a_1_-2d_4_-2d_2_ | 3a_2_+2d_2_-2d_3_ | 3a_3_+2d_4_+2d_3_ | 4 | 86.01 | 99.72 | 9.32 | 0.3 |
| 26 | | 3b_1_-2a_4_ | 3b_2_ | 3b_3_+2a_4_ | 4 | 92.50 | 106.22 | 15.82 | 0.0 |
| 27 | | 3b_1_-2b_4_-2b_2_ | 3b_2_+2b_2_ | 3b_3_+2b_4_ | 5 | 87.16 | 107.16 | 16.77 | 0.0 |
| 28 | | 3b_1_-2c_4_ | 3b_2_-2c_2_ | 3b_3_+2c_4_+2c_2_ | 5 | 85.39 | 105.39 | 14.99 | 0.0 |
| 29 | | 3b_1_-2d_4_-2d_2_ | 3b_2_+2d_2_-2d_3_ | 3b_3_+2d_4_+2d_3_ | 6 | 81.88 | 110.68 | 20.29 | 0.0 |
| 30 | | 3c_1_-2a_4_ | 3c_2_ | 3c_3_+2a_4_ | 3 | 87.52 | 96.52 | 6.12 | 1.6 |
| 31 | | 3c_1_-2b_4_-2b_2_ | 3c_2_+2b_2_ | 3c_3_+2b_4_ | 4 | 85.78 | 99.50 | 9.10 | 0.4 |
| 32 | | 3c_1_-2c_4_ | 3c_2_-2c_2_ | 3c_3_+2c_4_+2c_2_ | 4 | 87.46 | 101.18 | 10.78 | 0.2 |
| 33 | | 3c_1_-2d_4_-2d_2_ | 3c_2_+2d_2_-2d_3_ | 3c_3_+2d_4_+2d_3_ | 5 | 85.80 | 105.80 | 15.40 | 0.0 |
| 34 | | 3d_1_-2a_4_ | 3d_2_ | 3d_3_+2a_4_ | 4 | 85.46 | 99.17 | 8.77 | 0.4 |
| 35 | | 3d_1_-2b_4_-2b_2_ | 3d_2_+2b_2_ | 3d_3_+2b_4_ | 5 | 84.56 | 104.56 | 14.16 | 0.0 |
| 36 | | 3d_1_-2c_4_ | 3d_2_-2c_2_ | 3d_3_+2c_4_+2c_2_ | 5 | 86.33 | 106.33 | 15.94 | 0.0 |
| 37 | | 3d_1_-2d_4_-2d_2_ | 3d_2_+2d_2_-2d_3_ | 3d_3_+2d_4_+2d_3_ | 6 | 85.33 | 114.13 | 23.73 | 0.0 |
| 38 | | 3e_1_-2a_4_ | 3e_2_ | 3e_3_+2a_4_ | 3 | 87.52 | 96.52 | 6.13 | 1.6 |
| 39 | | 3e_1_-2b_4_-2b_2_ | 3e_2_+2b_2_ | 3e_3_+2b_4_ | 4 | 88.07 | 101.78 | 11.39 | 0.1 |
| 40 | | 3e_1_-2c_4_ | 3e_2_-2c_2_ | 3e_3_+2c_4_+2c_2_ | 4 | 88.33 | 102.04 | 11.64 | 0.1 |
| 41 | | 3e_1_-2d_4_-2d_2_ | 3e_2_+2d_2_-2d_3_ | 3e_3_+2d_4_+2d_3_ | 5 | 85.79 | 105.79 | 15.40 | 0.0 |
| *Vector-borne + Sex effect + Predation* | | | |  |  |  |  |  |  |
| 42 | | 3a_1_+1b_1_-2a_4_ | 3a_2_+1b_2_ | 3a_3_+1b_3_+2a_4_ | 5 | 76.66 | 96.66 | 6.26 | 1.5 |
| 43 | | 3a_1_+1b_1_-2b_4_-2b_2_ | 3a_2_+1b_2_+2b_2_ | 3a_3_+1b_3_+2b_4_ | 6 | 82.63 | 111.43 | 21.04 | 0.0 |
| 44 | | 3a_1_+1b_1_-2c_4_ | 3a_2_+1b_2_-2c_2_ | 3a_3_+1b_3_+2c_4_+2c_2_ | 6 | 74.48 | 103.28 | 12.89 | 0.1 |
| 45 | | 3a_1_+1b_1_-2d_4_-2d_2_ | 3a_2_+1b_2_+2d_2_-2d_3_ | 3a_3_+1b_3_+2d_4_+2d_3_ | 7 | 74.10 | 116.10 | 25.70 | 0.0 |
| 46 | | 3b_1_+1b_1_-2a_4_ | 3b_2_+1b_2_ | 3b_3_+1b_3_+2a_4_ | 7 | 78.80 | 120.80 | 30.41 | 0.0 |
| 47 | | 3b_1_+1b_1_-2b_4_-2b_2_ | 3b_2_+1b_2_+2b_2_ | 3b_3_+1b_3_+2b_4_ | 8 | 75.81 | 139.81 | 49.41 | 0.0 |
| 48 | | 3b_1_+1b_1_-2c_4_ | 3b_2_+1b_2_-2c_2_ | 3b_3_+1b_3_+2c_4_+2c_2_ | 8 | 73.41 | 137.41 | 47.01 | 0.0 |
| 49 | | 3b_1_+1b_1_-2d_4_-2d_2_ | 3b_2_+1b_2_+2d_2_-2d_3_ | 3b_3_+1b_3_+2d_4_+2d_3_ | 9 | 76.20 | 184.20 | 93.80 | 0.0 |
| 50 | | 3c_1_+1b_1_-2a_4_ | 3c_2_+1b_2_ | 3c_3_+1b_3_+2a_4_ | 6 | 74.29 | 103.09 | 12.70 | 0.1 |
| 51 | | 3c_1_+1b_1_-2b_4_-2b_2_ | 3c_2_+1b_2_+2b_2_ | 3c_3_+1b_3_+2b_4_ | 7 | 78.68 | 120.68 | 30.28 | 0.0 |
| 52 | | 3c_1_+1b_1_-2c_4_ | 3c_2_+1b_2_-2c_2_ | 3c_3_+1b_3_+2c_4_+2c_2_ | 7 | 72.83 | 114.83 | 24.43 | 0.0 |
| 53 | | 3c_1_+1b_1_-2d_4_-2d_2_ | 3c_2_+1b_2_+2d_2_-2d_3_ | 3c_3_+1b_3_+2d_4_+2d_3_ | 8 | 73.24 | 137.24 | 46.85 | 0.0 |
| 54 | | 3d_1_+1b_1_-2a_4_ | 3d_2_+1b_2_ | 3d_3_+1b_3_+2a_4_ | 7 | 78.18 | 120.18 | 29.78 | 0.0 |
| 55 | | 3d_1_+1b_1_-2b_4_-2b_2_ | 3d_2_+1b_2_+2b_2_ | 3d_3_+1b_3_+2b_4_ | 8 | 76.62 | 140.62 | 50.22 | 0.0 |
| 56 | | 3d_1_+1b_1_-2c_4_ | 3d_2_+1b_2_-2c_2_ | 3d_3_+1b_3_+2c_4_+2c_2_ | 8 | 74.11 | 138.11 | 47.71 | 0.0 |
| 57 | | 3d_1_+1b_1_-2d_4_-2d_2_ | 3d_2_+1b_2_+2d_2_-2d_3_ | 3d_3_+1b_3_+2d_4_+2d_3_ | 9 | 74.67 | 182.67 | 92.27 | 0.0 |
| 58 | | 3e_1_+1b_1_-2a_4_ | 3e_2_+1b_2_ | 3e_3_+1b_3_+2a_4_ | 6 | 73.52 | 102.32 | 11.92 | 0.1 |
| 59 | | 3e_1_+1b_1_-2b_4_-2b_2_ | 3e_2_+1b_2_+2b_2_ | 3e_3_+1b_3_+2b_4_ | 7 | 76.92 | 118.92 | 28.52 | 0.0 |
| 60 | | 3e_1_+1b_1_-2c_4_ | 3e_2_+1b_2_-2c_2_ | 3e_3_+1b_3_+2c_4_+2c_2_ | 7 | 74.52 | 116.52 | 26.12 | 0.0 |
| 61 | | 3e_1_+1b_1_-2d_4_-2d_2_ | 3e_2_+1b_2_+2d_2_-2d_3_ | 3e_3_+1b_3_+2d_4_+2d_3_ | 8 | 73.74 | 137.74 | 47.34 | 0.0 |
| *Environmental* | |  |  |  |  |  |  |  |  |
| 62 | | 3f_1_ | 3f_2_ | 3f_3_ | 3 | 86.84 | 95.84 | 5.44 | 2.3 |
| *Environmental + Predation* | | |  |  |  |  |  |  |  |
| 63 | | 3f_1_+1b_1_ | 3f_2_+1b_2_ | 3f_3_+1b_3_ | 6 | 75.68 | 104.48 | 14.08 | 0.0 |

**Appendix S2.** North American felids singly infected with *CMhm* or co-infected with *CMhm* and either *M. haemofelis* or “*Ca.* M. turicensis”.

| **SINGLE INFECTIONS** | |  |  |  |  |
| --- | --- | --- | --- | --- | --- |
|  | **NLA** | **SLA** | **FR** | **WS** | **TOTAL** |
| **Puma** | 2 | 5 | 4 | 4 | **15** |
| **Bobcat** | 8 | 3 | 5 | 1 | **17** |
| **Domestic Cat** | 5 | 5 | 3 | 2 | **15** |
| **TOTAL** | **15** | **13** | **12** | **7** | **47** |
|  |  |  |  |  |  |
| **CO-INFECTIONS** |  |  |  |  |  |
|  | **NLA** | **SLA** | **FR** | **WS** | **TOTAL** |
| **Puma** | 0 | 0 | 1 | 1 | **2** |
| **Bobcat** | 5 | 0 | 0 | 2 | **7** |
| **Domestic Cat** | 2 | 1 | 0 | 1 | **4** |
| **TOTAL** | **7** | **1** | **1** | **4** | **13** |

**Appendix S3.** All samples used throughout analyses. ‘Newly characterized samples’ were used for modeling transmission mechanisms; all samples were used for phylogenetic analyses. FR = front range; WS = western slope; NLA = north of Los Angeles; SLA = south of Los Angeles. New samples will be updated with GenBank accession numbers upon acceptance of manuscript.

| **Newly Characterized Samples** |  |  |  |  |  |
| --- | --- | --- | --- | --- | --- |
| **Animal ID** | **No. Samples** | **Host Species** | **Capture Location** | **Region** |  |
| X1030; X1217; X1325 | 3 | *F. catus* | USA | CO-FR |  |
| X1499; X596; X587* | 3 | *F. catus* | USA | CO-WS |  |
| X1001; X1062; X1209; X1314; X706; X1315*; X702* | 7 | *F. catus* | USA | CA-NLA |  |
| X1000; X1140; X1239; X1503; X1126*; X672 | 6 | *F. catus* | USA | CA-SLA |  |
| X1288; X1313; X1316; X1328; X1363 | 5 | *L. rufus* | USA | CO-FR |  |
| X947; X364*; X937* | 3 | *L. rufus* | USA | CO-WS |  |
| X1299; X1300; X1301; X1302; X1303; X1509; X1510; X905; X1537*; X913R1*; X192*; X1513*; X1304* | 13 | *L. rufus* | USA | CA-NLA |  |
| X1065; X384; X599 | 3 | *L. rufus* | USA | CA-SLA |  |
| X1054; X1064; X1076; X433R1; X1346* | 5 | *P. concolor* | USA | CO-FR |  |
| X1058; X1131; X403; X686*; X224 | 5 | *P. concolor* | USA | CO-WS |  |
| X1582; X1591 | 2 | *P. concolor* | USA | CA-NLA |  |
| X1207; X393; X704; X871; X952R1 | 5 | *P. concolor* | USA | CA-SLA |  |
| **Total** | **60** |  |  |  |  |
|  |  |  |  |  |  |
| **Previously Described Samples** |  |  |  |  |  |
| **GenBank Accession No.** | **No. Samples** | ***Species*** | **Origin** |  |  |
| AM745338 | 1 | *F. catus* | China | . |  |
| AY150974 | 1 | *F. catus* | Israel |  |  |
| AY150979 | 1 | *F. catus* | South Africa | | |
| AY150980; HE613254; AY150981 | 3 | *F. catus* | UK |  |  |
| AY529634 | 1 | *F. catus* | Japan |  |  |
| DQ157141; DQ157142; DQ157143; DQ157144; DQ157145; DQ157146; DQ157147; DQ157148; | 8 | *F. catus* | Switzerland |  |  |
| EU128752 | 1 | *F. catus* | Hungary |  |  |
| EU839979; EU839980; EU839981; EU839982; EU839983; EU839984; EU839985 | 7 | *F. catus* | Italy |  |  |
| FJ004275; KF743737; KF743738; KF743739; U88564 | 5 | *F. catus* | USA |  |  |
| KM275248; KM275249; KM275250; KM275251; KM275252; KM275253; KM275254; KM275255; KM275256 | 9 | *F. catus* | Brazil |  |  |
| EU285281 | 1 | *F. catus* | Thailand |  |  |
| DQ825442; DQ825443 | 2 | *F. silvestris* | France |  |  |
| DQ825444; DQ825445; DQ825446 | 3 | *Linx. Pardinus* | Spain |  |  |
| DQ825452; DQ825453 | 2 | *Panhero leo* | Tanzania |  |  |
| DQ825440 | 1 | *L. wiedii* | Brazil |  |  |
| DQ825439 | 1 | *L. tigrinus* | Brazil |  |  |
| DQ825456; DQ825457 | 2 | *L. lynx* | Switzerland |  |  |
| AF338269 ** | 1 | *S. sciureus* |  |  |  |
| **Total** | **50** |  |  |  |  |

**Appendix S4. Details of PCR amplification, sequencing, detection of co-infections, and phylogenetics**

*PCR amplification*

Genomic DNA was extracted from whole blood using the QIAamp DNeasy blood and tissue kit (Qiagen Inc., Valencia, CA). We amplified the 16S rRNA gene with two pairs of forward and reverse PCR primers used in previous studies (Pitulle *et al.* 1999; Criado-Fornelio *et al.* 2003; Barker 2011) with a single nucleotide modification to the [Pitulle et al. (1999](#_ENREF_69)) 8F universal primer. These primers amplified a total of 1484 nucleotides with an overlap of 595 base pairs (Appendix 3).

PCR methodology was adapted from [Criado-Fornelio et al. (2003](#_ENREF_23)) with the substitution of HotStarTaq DNA polymerase (Qiagen Inc.) in place of Amplitaq Gold DNA polymerase (Applied Biosystems, Inc., Foster City, CA) for most samples. Reaction mixtures (25 μL) contained 12.5 μL HotStarTaq, 9 μL sterile-filtered PCR water, 0.5 μL of each forward and reverse primer, and 2.5 μL DNA. For some samples, the master mix was doubled from 25 to 50 μL; all components of reaction mixtures were likewise doubled for these samples. We used a GeneAmp PCR System 9700 PCR thermocycler (Applied Biosystems) for all amplifications. Having determined optimal primer annealing temperature using an annealing gradient, the final thermocycling profile was as follows: 94 **°**C for 10 min followed by 40 cycles of (95 **°**C for 30 sec; 52 °C for 30 sec; 72 °C for 60 sec), followed by 72 °C for 10 min. All PCR products as well as positive and negative controls were visualized under UV light by gel electrophoresis using 1.5% agarose gel and EZ-Vision 6x DNA dye (Amresco, Solon, OH). Positive controls included samples that previously tested positive for *CMhm*; negative controls were sterile water.

*Sequencing and alignment*

PCR products were purified prior to sequencing using either the QIAquick Gel Extraction Kit or QIAquick PCR Purification Kit (Qiagen Inc.) depending on the presence or absence of multiple bands, respectively. DNA concentrations varied widely among samples, yielding between 10 and 120 ng/μL. Samples were directly sequenced in both directions in bulk at the University of Chicago Comprehensive Cancer Center; when fewer than 12 samples were sequenced at one time, we used the Proteomics and Metabolomics Facility at Colorado State University. We accepted sequences with ≥ 2x high-quality coverage and upon visual examination using Geneious version 7.1. (http://www.geneious.com, Kearse *et al.* 2012). Additionally, we identified in GenBank another 49 previously described *Candidatus* Mycoplasma haemominutum (*CMhm*) sequences, as well as a *Candidatus* Mycoplasma kahanei sequence (host: *Saimiri sciureus*) for use as an outgroup in our phylogenetic analysis (Appendix 2). We aligned all sequences using MAFFT version 7 method Q-INS-I (Katoh & Toh 2008) and trimmed all sequences at the 5’ and 3’ ends such that all sequences were the same length (1238 bp). We aligned each sequence to the 16S rRNA sequence extracted from the noncontiguous finished genome sequence of *CMhm* (GenBank accession no. HE613254).

*Co-infections*

We discovered a high rate of multiply-infected samples by closely inspecting what appeared to be low quality reads containing long sequences with double- and triple-peaked chromatograms. Upon examination, we determined these chromatograms arose from co-infected individuals that carried two or more sequences that differed from each other because of indels. We compared alignments of sequence data from singly-infected individuals against the putative co-infected samples, and found that in most cases, frameshifts were clear and their positions corresponded with loci at which indels occurred in other sequences. Upon inspecting the sequences manually, we discovered that most putatively co-infected sequences with indel mutations realigned with known sequences when accounting for missing nucleotides. Subsequently, all reads with > 1 peak at any given nucleotide locus were visually examined for frameshifts indicative of multiple *CMhm* infections.

We then automated and streamlined the process used to identify the individual haplotypes in co-infected sequences using 1) Mixed Sequence Reader (Chang *et al.* 2012) to extract two distinct sequences from double-peaked chromatograms, 2) Geneious 7.1.5 (Kearse *et al.* 2012) to visually verify all heterozygous base calls, 3) SeqPHASE (Flot 2010) to format our data, and finally 4) the program PHASE (Stephens & Donnelly 2003) to reconstruct co-infected haplotypes. In PHASE, we ran 10,000 iterations with a thinning interval of 5 and a burn-in of 100. We used the original mutation model without recombination or stepwise mutation for multi-allelic loci (Stephens, Smith & Donnelly 2001).

*Phylogenetics*

For phylogenetic analyses, we used our complete dataset of 73 novel North American sequences as well as 49 previously described sequences available from GenBank (Figure 1b; Appendix 2). We used the BEAUTi graphical user interface for program BEAST version 1.8.4 to input parameters for Bayesian Markov chain Monte Carlo (MCMC) analyses (Drummond & Rambaut 2007; Drummond *et al.* 2012), making use of the BEAGLE library to improve computational performance (Suchard & Rambaut 2009). We combined three MCMC chains of 1.2E8 iterations, discarding the first 10% as burn-in and recording parameters every 12000 trees. In jModelTest2 (Darriba *et al.* 2012), we used AIC to select the generalized time reversible model with both invariable sites and gamma-distributed rate variation among sites (GTR+I+G). We used default priors (Appendix S6) and assumed a coalescent model with a strict molecular clock, as we lack information on long-term mutation rates with which to parameterize a different clock. We assumed a constant bacterial population size over time because of the clonal nature of *CMhm* and its endemism in felids. Nodes of the tree were estimated using substitutions per site, with node probabilities derived from the posterior distribution (PP).

We accepted the final MCC tree upon ensuring convergence of parameter estimates and effective sample sizes (ESS) of > 200, where the ESS represents a measurement of adequate mixing of parameters and a lack of auto-correlation between states. The MCC represents a model-averaged tree in which the contribution of each sampled tree to the final model is proportional to its posterior likelihood. We used the program Tracer version 1.6 (Rambaut & Drummond 2013) to calculate all marginal posterior probabilities and measures of central tendency from the posterior distribution of parameters. We superimposed all tree elements onto our MCC tree using FigTree version 1.4.2 (Rambaut 2007).

**Appendix S5.**

Primers used for amplification of 16S rDNA. Nucleotide position numbers correspond to base pair positions within the fully sequenced genome of *CMhm* (Barker *et al.* 2012), GenBank Accession No. HE613254. Each fragment was sequenced in both directions using each primer pair to ensure that all nucleotides were called at least twice.

| **Primer** | **Direction** | **Sequence** | **Nucleotide Position** | **Amplified Fragment Length** | **Source** |
| --- | --- | --- | --- | --- | --- |
| 8F | Forward | 5'-AGAGTTTGATCCTGGCTCAG-3' * | 306,274 | 933 | Pittule et al. 1999 |
| 908R | Reverse | 5'-TGCTCCACCACTTGTTCA-3' | 305,361 | 933 | Criado-Fornelio et al. 2003 |
| 313F | Forward | 5'-ATACGGCCCATATTCCTACG-3' | 305,960 | 1268 | Criado-Fornelio et al. 2003 |
| 1492R | Reverse | 5'-GGTTACCTTACGACTT-3' | 304,834 | 1268 | Pitulle et al. 1999 |

* modified for our study from the original published version by a single nucleotide (T-C) in the 11^th^ position (5’-3’ direction)

**Appendix S6.** For all phylogenetic reconstructions using BEAST v1.8.4 (Drummond & Rambaut 2007; Drummond *et al.* 2012) on 16S rRNA sequences, the following default weak priors were used for the GTR + Γ + I model and asymmetrical host and location state transition rate estimation in BEAST:

- A-C substitutions ~ gamma(0.05, 10), initial value = 1
- A-G substitutions ~ gamma(0.05, 20), initial value = 1
- A-T substitutions ~ gamma(0.05, 10), initial value = 1
- C-G substitutions ~ gamma(0.05, 10), initial value = 1
- G-T substitutions ~ gamma(0.05, 10), initial value = 1
- Base frequences ~ uniform(0, 1), initial value = 0.25
- Gamma shape parameter ~ exponential(0.5), initial value = 0.5
- Proportion of invariant sites ~ uniform(0, 1), initial value = 0.5
- Number of non-zero host state transition rates for BSSVS ~ Poisson(9)
- Host state frequencies ~ uniform(0, 1), initial value = 0.25
- Host state instantaneous transition rates ~ gamma(1, 1), initial value = 1
- Number of non-zero location state transition rates for BSSVS ~ Poisson(14)
- Location state frequencies ~ uniform(0, 1), initial value = 0.25
- Location state instantaneous transition rates ~ gamma(1, 1), initial value = 1

**Appendix S7.** Neighbor-joining (A), UPGMA (B), and Maximum-Likelihood (C) trees reinforcing the topology of our Bayesian ultrametric tree. Neighbor-joining and UPGMA trees created with MEGA using the Tamura-Nei sequence evolution model; node support estimated using 1000 bootstrap replicates. Maximum-Likelihood tree created with RAxML with the GTR+G+I model with 1000 bootstrap replicates. Tip names correspond to samples listed in Appendix S3.

**(A)**

**
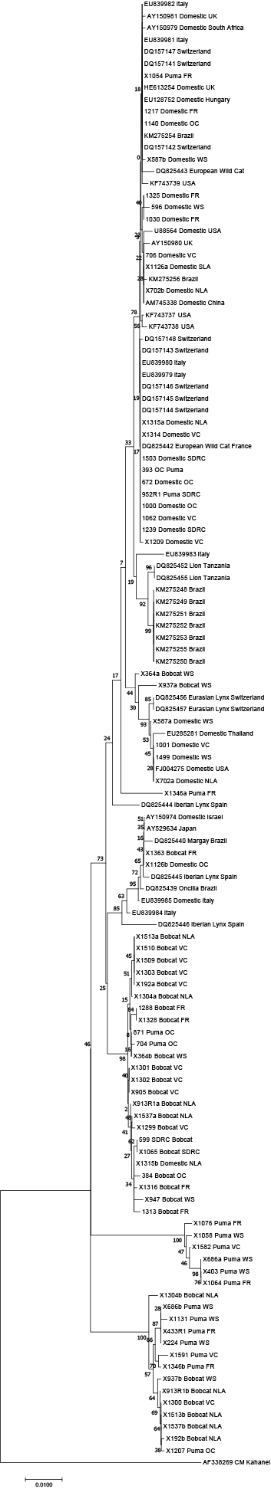
**

**(B)**

**
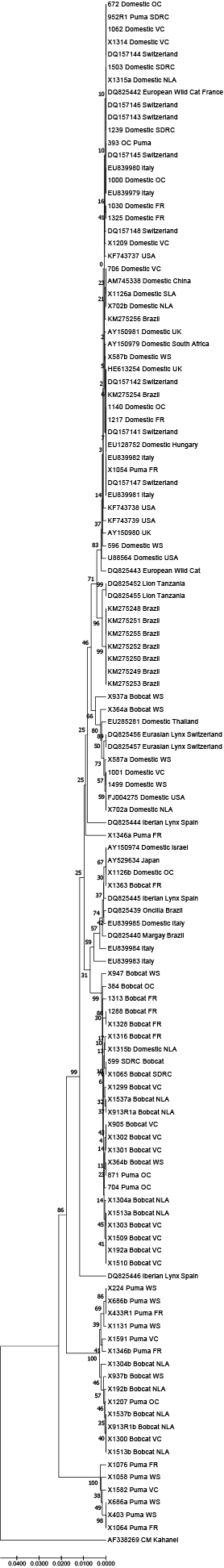
**

**(C)**

**
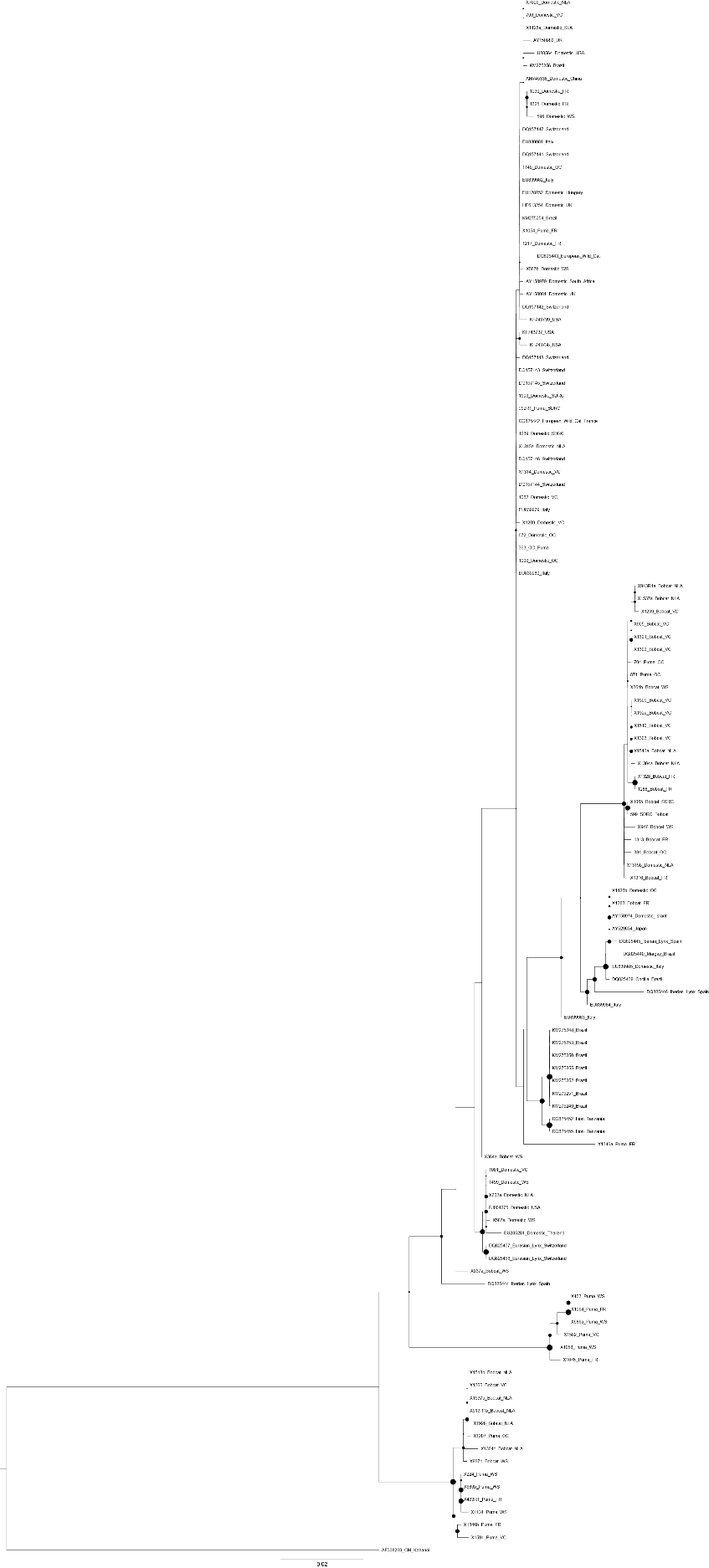
**

**REFERENCES**

Barker, E. (2011) *Haemotropic Mycoplasmas of Mammals*. University of Bristol, Langford House, Bristol BS40 5DU, United Kingdom.

Barker, E.N., Darby, A.C., Helps, C.R., Peters, I.R., Hughes, M.A., Radford, A.D., Novacco, M., Boretti, F.S., Hofmann-Lehmann, R. & Tasker, S. (2012) Genome sequence for “Candidatus Mycoplasma haemominutum,” a low-pathogenicity hemoplasma species. *J Bacteriol*, **194**, 905–906.

Chang, C.T., Tsai, C.N., Tang, C.Y., Chen, C.H., Lian, J.H., Hu, C.Y., Tsai, C.L., Chao, A., Lai, C.H., Wang, T.H. & Lee, Y.S. (2012) Mixed Sequence Reader: A Program for Analyzing DNA Sequences with Heterozygous Base Calling. *Scientific World Journal*.

Criado-Fornelio, A., Martinez-Marcos, A., Buling-Sarana, A. & Barba-Carretero, J.C. (2003) Presence of mycoplasma haemofelis, mycoplasma haemominutum and piroplasmids in cats from southern europe: A molecular study. *Veterinary microbiology*, **93**, 307–317.

Darriba, D., Taboada, G.L., Doallo, R. & Posada, D. (2012) jModelTest 2: more models, new heuristics and parallel computing. *Nature Methods*, **9**, 772.

Drummond, A.J. & Rambaut, A. (2007) BEAST: Bayesian evolutionary analysis by sampling trees. *BMC evolutionary biology*, **7**, 214.

Drummond, A.J., Suchard, M.A., Xie, D. & Rambaut, A. (2012) Bayesian phylogenetics with BEAUti and the BEAST 1.7. *Molecular Biology and Evolution*, **29**, 1969–1973.

Flot, J.F. (2010) SEQPHASE: a web tool for interconverting phase input/output files and fasta sequence alignments. *Molecular Ecology Resources*, **10**, 162–166.

Katoh, K. & Toh, H. (2008) Recent developments in the MAFFT multiple sequence alignment program. *Briefings in Bioinformatics*, **9**, 286–298.

Kearse, M., Moir, R., Wilson, A., Stones-Havas, S., Cheung, M., Sturrock, S., Buxton, S., Cooper, A., Markowitz, S., Duran, C., Thierer, T., Ashton, B., Meintjes, P. & Drummond, A. (2012) Geneious Basic: An integrated and extendable desktop software platform for the organization and analysis of sequence data. *Bioinformatics*, **28**, 1647–1649.

Pitulle, C., Citron, D.M., Bochner, B., Barbers, R. & Appleman, M.D. (1999) Novel bacterium isolated from a lung transplant patient with cystic fibrosis. *Journal of Clinical Microbiology*, **37**, 3851–3855.

Rambaut, A. (2007) FigTree, a graphical viewer of phylogenetic trees. *See http://tree. bio. ed. ac. uk/software/figtree*.

Rambaut, A. & Drummond, A. (2013) Tracer 1.6. University of Edinburgh, Edinburgh, UK.

Stephens, M. & Donnelly, P. (2003) A comparison of Bayesian methods for haplotype reconstruction from population genotype data. *American Journal of Human Genetics*, **73**, 1162–1169.

Stephens, M., Smith, N.J. & Donnelly, P. (2001) A new statistical method for haplotype reconstruction from population data. *American Journal of Human Genetics*, **68**, 978–989.

Suchard, M.A. & Rambaut, A. (2009) Many-core algorithms for statistical phylogenetics. *Bioinformatics*, **25**, 1370–1376.
